# Annual Report 2021

**DOI:** 10.3934/microbiol.2022009

**Published:** 2022-03-28

**Authors:** Xu Guo

**Affiliations:** Room 703, Luoke Times Center, Anli Road, Chaoyang District, Beijing 100101, China

## 1. Journal summary

AIMS Microbiology is an international Open Access journal devoted to publishing peer-reviewed, high quality, original papers in the field of microbiology. Together with the Editorial Office of AIMS Microbiology, I wish to testify my sincere gratitude to all authors, members of the editorial board and reviewers for their contribution to AIMS Microbiology in 2021.

The manuscript submissions to our journal in 2021 increased significantly. We had more than 200 submitted manuscripts and 32 of them were accepted and published, of these 23 were research articles and 9 were review articles. The authors of the manuscripts are from more than 15 countries, about 50% of them from developed countries and the rest from developing countries. The data shows a significant increase of international collaborations on the research of microbiology.

An important part of our strategy has been preparation of special issues. One special issue published more than five papers. AIMS Microbiology have invited 15 experts to join our Editorial Board in 2021. We will continue to renew Editorial Board in 2022.

We hope that in 2022, with the support of all the members of the editorial board and reviewers, the journal can receive and collect more excellent articles to be able to publish. The journal will dedicate to publishing high quality papers by regular issues as well as special issues organized by the members of the editorial board. All these efforts will increase the impact and citations of the papers to significantly increase the academic rank and impact of AIMS Microbiology as much as possible.

*On behalf of*

*AIMS Microbiology Editorial Board*

## 2. Editorial development

### 2.1. Manuscript statistics

The submissions of our AIMS Microbiology journal in 2021 increased. In 2021, AIMS Microbiology published 4 issues, a total of 32 articles were published online, and the category of published articles is as follows:

**Table:** 

Type	Number
Research	23
Review	9

**Figure:**
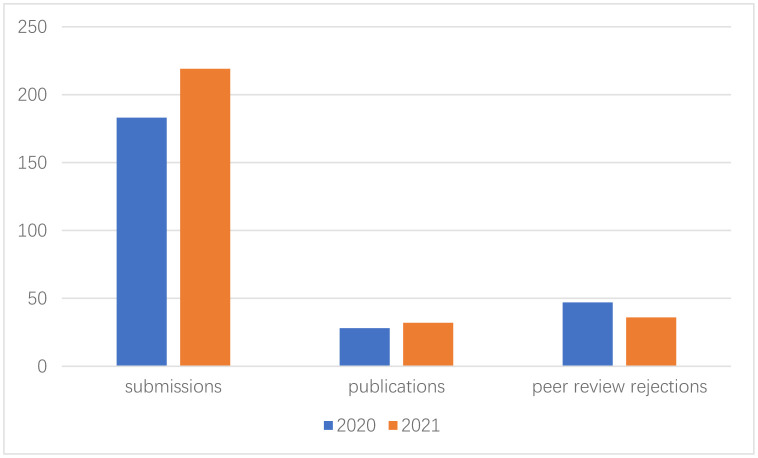


Peer Review Rejection rate: 47%

Publication time (from submission to online): 75 days

### 2.2. Author distribution

The authors' country of origin distribution is shown below. It is pleased to see that so many diversified countries were represented among our authors.

**Figure:**
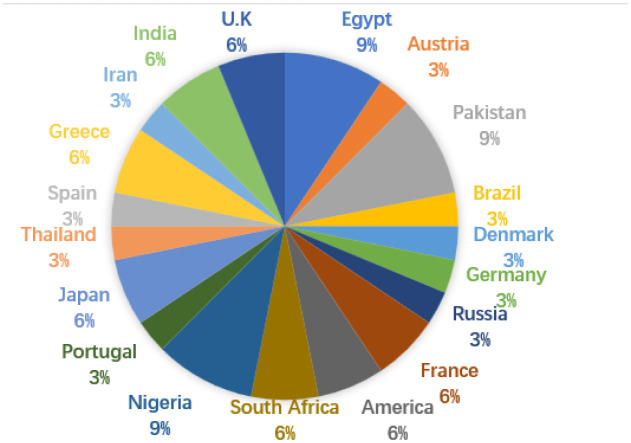


### 2.3. Editorial Board members

AIMS Microbiology has Editorial Board members representing researchers from 19 countries, which are shown below. We are constantly assembling the editorial board to be representative to a variety of disciplines across the field of microbiology. AIMS Microbiology has 81 members now, and 15 of them joined in 2021. We will continue to invite dedicated experts and researchers in order to renew the Editorial Board in 2022.

**Figure:**
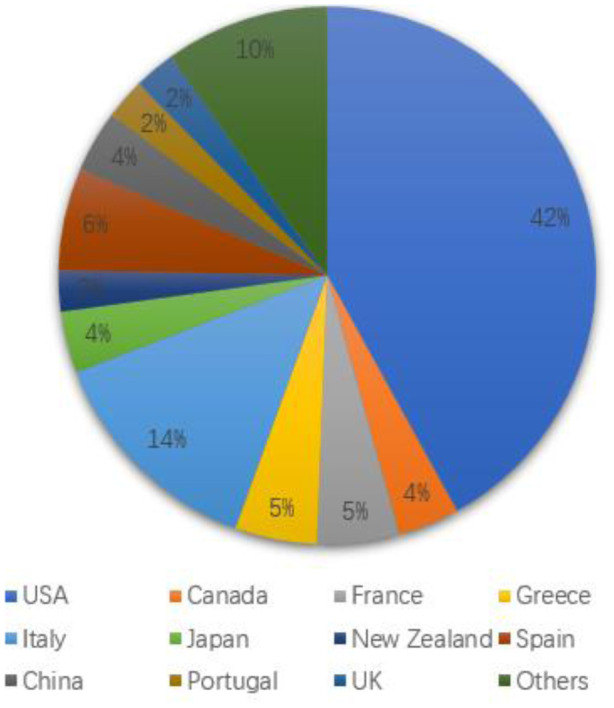


### 2.4. Articles metrics

Top 10 Cited Papers in last 2 years.

**Table:** 

No.	Article	Citations
1	Saccharomyces cerevisiae and its industrial applications	50
2	Six decades of lateral flow immunoassay: from determining metabolic markers to diagnosing COVID-19	25
3	Molecular docking between human TMPRSS2 and SARS-CoV-2 spike protein: conformation and intermolecular interactions	18
4	Exopolysaccharide production by lactic acid bacteria: the manipulation of environmental stresses for industrial applications	13
5	Biologically active compounds from marine organisms in the strategies for combating coronaviruses.	7
6	Vitamin D level and it is association with the severity of pulmonary tuberculosis in patients attended to Kosti Teaching Hospital, Sudan	6
7	Insights of Novel Coronavirus (SARS-CoV-2) disease outbreak, management and treatment	5
8	An in silico analysis of acquired antimicrobial resistance genes in Aeromonas plasmids	5
9	Selective enrichment of heterotrophic nitrifiers Alcaligenaceae and Alcanivorax spp. from industrial wastewaters	5
10	Emerging superbugs: The threat of Carbapenem Resistant Enterobacteriaceae	3

### 2.5. Summary & plan

#### 2.5.1. Summary

In the recent two years, our journal has developed much faster than before; Our journal has been indexed in Web of Science, Scopus and PubMed databases. We received more than 200 manuscript submissions and published 32 papers in 2021. We have added 15 new Editorial Board members.

#### 2.5.2. Plan in 2022

In 2022, we expect to publish more and better articles to enhance the reputation of the journal. We will invite more experts in the field of microbiology to publish a review or research article. To set a goal, we would like to publish 40 high-quality articles in 2022. We hope Editorial Board members could help us invite some reputational scholars in your network to contribute articles to our journal. We also would like to invite our board members to try to increase the influence and impact of AIMS Microbiology by soliciting and advertising high quality articles and special issues.

